# In or Out?

**Published:** 1906-03

**Authors:** 


### EDITORIAL NOTES AND COMMENTS.

The Business office of THE JOURNAL-RECORD is No. 1007-1008 Century Building.

The Editorial office is 318-319-320 The Prudential.

Address all Business communications to Dr. M. B. Hutchins, Mgr.

Make remittances payable to THE ATLANTA JOURNAL-RECORD OF MEDICINE.

On matters pertaining to the Editorial and Original Communications address Dr. Bernard Wolff, Atlanta.

Reprints of original articles will be furnished at cost price. Requests for the same should always be made *on the manuscript*.

We will present, post-paid, on request, to each contributor of an original article, twenty (20) marked copies of THE JOURNAL-RECORD containing such article.

### IN OR OUT?

URBAN dwellers, where the surrounding country does not produce sufficient for their physical needs, and who have them supplied from a distance, will be interested in the discussion now going on in sanitary circles upon the question of drawn or undrawn poultry and fish. It is contended by some that the act of drawing (which is the euphemistic word for gutting or evisceration) hastens putrefaction by affording an atrium for the entrance of putrefactive micro-organisms. The opponents of this hold that as soon as the fowl or fish is dead putrefaction begins, and that the intestines are the first concerned in the process. The result is that the flesh is permeated with the products of decomposition of not only the intestines themselves, but of their contents. This flavor may add a piquancy to the flesh as food which is highly prized by some but not by others. The Department of Agriculture has conducted some experiments in this direction which led to the discovery that under the same conditions of temperature and humidity a drawn fowl will keep from twenty to thirty days longer than one which is not drawn. Bulletin No. 144 of the Department remarks that it is admitted that opening of the body of an animal and exposing the internal surface to the air may have some influence in hastening putrefaction, but if the drawing process is properly conducted, without rupture of the intestines and escape of their contents, and the cavity washed out and rubbed with salt, the objection to removing the viscera is set aside.

The ancient Egyptians appeared to realize that the abdominal contents were first concerned in the act of putrescence and on that account in the preparation of mummies evisceration was practiced. The modern cold-storage fowl, minus the cere cloths, might with some justification be compared to a mummy. The date of death of each, at least, is indeterminate. It is not pleasant to think that we are feasting on the body of a fowl which cackled cheerily in the heat of the western plains last summer or the summer before last, and it is assuredly the part of a protective government to make things as smooth as possible for its confiding subjects. In this age of investigation it seems but proper that this spirit would even pervade the penetralia of a hen.

